# Rapid assessment of the authenticity of limequat fruit using the electronic nose and gas chromatography coupled with mass spectrometry

**DOI:** 10.1007/s00706-018-2242-7

**Published:** 2018-08-09

**Authors:** Martyna Lubinska-Szczygeł, Dominika Pudlak, Tomasz Dymerski, Jacek Namieśnik

**Affiliations:** Department of Analytical Chemistry, Faculty of Chemistry, Gdańsk University of Technology, 11/12 G. Narutowicza Str., 80-233 Gdańsk, Poland

**Keywords:** Electronic nose, Fragrances, Gas chromatography, Natural products, Hybrid fruits

## Abstract

**Abstract:**

Citrus fruits are very popular food products. There are many species and varieties of them. There are also documented cases of some citrus fruits causing a severe allergic reaction. Some species of the citrus fruits, especially hybrid ones show a reduced allergenic effect due to the lack of seeds. There is a need for rapid methods for evaluation of citrus’ botanical origin. During research, the headspace of three citrus fruits *Citrus Aurantifolia, Citrus japonica*, and *Citrus *× *floridana* was analysed using electronic nose based on ultrafast gas chromatography and gas chromatography with mass spectrometry. In the paper, two approaches were compared. The usefulness of an electronic nose to control the quality of hybrids was demonstrated. The results obtained during ultrafast gas chromatography analyses were subjected to statistical analysis. Four chemometric methods namely: principal component analysis (PCA), discriminant function analysis (DFA), soft independent modeling of class analogies (SIMCA), statistical quality control (SQC) were used to distinguish between limequat and its parent fruits. Electronic nose combined with chemometrics is a novel analytical tool for hybrid fruits’ classification due to their botanical origin. It can supplement established techniques by providing results in a short time and at a low cost.

**Graphical abstract:**

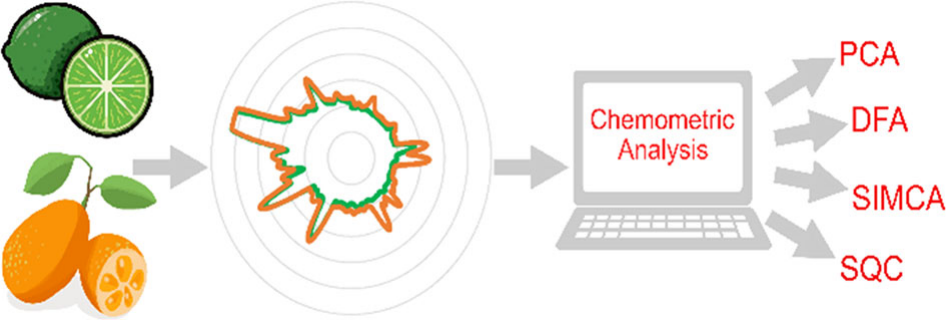

## Introduction

Citruses are the most popular group of fruits in the world. Citrus fruit production in the world in the 2016/2017 season was 49.3 million tons. United States and Brazil are the main fruit producers [1]. It is concerned that the south-east of Asia is the place of origin of citrus fruits [2]. In the Asian region, there are many varieties of citrus fruit that have distinctive flavour characteristics [3]. New citrus varieties are increasingly being produced as a result of hybridization. Most of the commonly available and consumed citrus fruits, among others like lemon, orange or lime are hybrid fruits. The parent fruits include pummelo, mandarin, citron, and Australian lime. Hybrid fruits combine the properties of parent fruits, while not having their disadvantages. The next ones in the hybrid chain become therefore more and more perfect in terms of nutritional values, functional traits, but mainly organoleptic properties. There is a large number of fruit species and varieties within one genus. Lots of them look similar and in some cases, based on visual assessment, it is not possible to recognize the fruit variety. It is sometimes hard to distinguish citruses from their parent fruit, because hybrid fruits inherit their visual characteristics. Misidentifications of citrus variety occurs commonly, especially in the case of limes and grapefruit varieties, because, visually, there are only small differences between them resulting from different shapes or colours. Knowledge of botanical origin is particularly important from the point of view of potential allergies [4]. Allergens contained in citrus are among the most common factors causing allergies [5]. The content of allergens is different for various citrus fruits, which is why it is extremely important to know the botanical origin of the fruit. The most allergenic factor in citrus is their seeds, which contain numerous IgE reactive proteins [6]. Citrus hybrids are often seedless, hence their reduced potency of the allergenic effect.

Therefore, some new solutions to assess the botanical origin of fruits are considered. Gas chromatography is a popular analytical technique used for this purpose. Hong et al. used multidimensional gas chromatography (MDGC) to evaluate the aroma profile of *Citrus junos Sieb. ex Tanaka* (yuzu), *Citrus limon BURM*. *f*. (lemon), and *Citrus aurantifolia Christm.* Swingle (lime). They demonstrated the utility of this technique to determine the botanical origin of fruits based on the analysis of the volatile chiral compounds, i.e., α-pinene, camphene, sabinene, limonene, and β-phellandrene [7]. The disadvantage of this solution is the time taken for a single analysis [8]. In the case of MDGC, it lasts for several dozens of minutes. Chromatographic techniques also require the identification of chemical compounds, e.g., by comparing the retention time of the analyte and the reference substance, which generates high costs of analysis [9]. Therefore, there is a need for solutions that make it possible to distinguish hybrid fruits in the shortest possible time, without complicated analytical procedures. A good solution for distinguishing citrus fruit can be an electronic nose based on ultrafast gas chromatography (UFGC). The main benefits of using UFGC technique is relatively short time of single analysis, which increases the throughput of the laboratory and significantly reduces costs. In this equipment, a fingerprint technique is used to distinguish between the samples, which allows the analysis of headspace without prior chromatographic separation [10].

The aim of the study was to assess the usefulness of gas chromatography coupled with time-of-flight mass spectrometry and the electronic nose based on ultrafast gas chromatography to distinguish the hybrid fruit regarding their parent fruits, namely lime and kumquat. Two approaches, concerning fingerprint analysis and classical chromatographic analysis, were applied. There are many scientific reports about using electronic nose to distinguish between fruit species. However, hybrid fruits volatile fraction is very difficult to analyse due to the similarities to parent fruit. To the best of our knowledge, the existing body of literature on this subject matter is not exhausted yet.

## Results and discussion

**Fig. 1:**
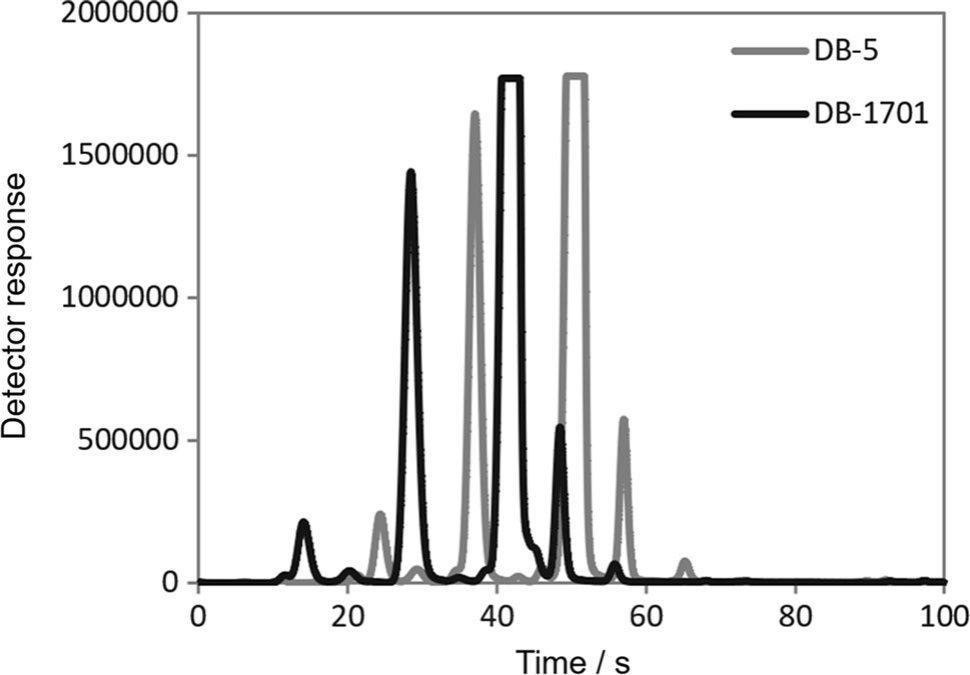
Chromatogram obtained by performing an analysis of limequat sample using ultra-fast GC-FID technique

During the research of the headspace of the kumquat, limequat, and lime fruits two apparatus were used. The first of them was the ultrafast gas chromatograph coupled with a μFID detector. This instrument allows to conduct short chromatographic analyses (in the presented studies, the time of a single analysis was established to 90 s). This time may be insufficient to separate most of the analytes present in samples, but in the case of classification it is not necessary. In Table 1, 20 major, tentatively identified chemical compounds, were detected in the citrus fruits by the use of ultrafast GC. It can be noticed, the terpenes were most abundant among the identified volatile compounds of kumquat, limequat, and lime fruits. This information is consistent with the literature data. Güney et al. studied the composition of the volatile fraction of different kumquat varieties, and based on the results, they found that terpenes were min. 87% of kumquat volatile fraction and about 98% of the area of all chromatographic peaks detected in the limequat headspace [11]. Lubinska-Szczygieł et al. proved that terpenes are min. 53% of all volatile substances present in the key lime [12]. However, it should be remembered that using the ultrafast GC technique only the tentative identification of detected peaks is possible. This identification is based only on the comparison of Kovats retention indexes for the respective signals. A typical chromatogram obtained by analysing limequat sample with the use of ultra-fast GC is presented in Fig. 1.

**Table 1:** Tentative identification of major volatiles present in kumquat, limequat, and lime provided by the use of ultra-fast GC-FID technique

No.	Compound	Relative peak area mean ± SD/(%)		
		Kumquat	Limequat	Lime
1	α-Terpinene	40.16 ± 0.39^a,b^	25.5 ± 2.4^a^	24.51 ± 0.28^b^
2	α-Phellandrene	41.4 ± 3.4^b^	33.2 ± 2.4^c^	24.67 ± 0.22^b,c^
3	2-Heptenal	n.d.	12.1 ± 2.2^c^	5.00 ± 0.42^c^
4	3-Heptanone	2.74 ± 0.42^a^	12.2 ± 2.1^a^	4.87 ± 0.41^a^
5	Limonene	0.355 ± 0.053^b^	3.40 ± 0.50	4.15 ± 0.48^b^
6	γ-Terpinene	0.475 ± 0.036^b^	2.87 ± 0.43	4.10 ± 0.48^b^
7	2-Heptanone	0.482 ± 0.033	1.71 ± 0.35	0.976 ± 0.087
8	Ethyl isovalerate	0.57 ± 0.14	1.71 ± 0.35	0.974 ± 0.086
9	δ-Decalactone	n.d.	0.319 ± 0.027	0.1331 ± 0.0064
10	Cymenene	0.222 ± 0.022	0.525 ± 0.070	0.459 ± 0.027
11	2.4-Hexadienal	0.0045 ± 0.0013	0.473 ± 0.017	0.275 ± 0.019
12	Hexadecane	0.0415 ± 0.0031	0.388 ± 0.011	0.1973 ± 0.0099
13	Isoamyl acetate	0.0052 ± 0.0011	0.381 ± 0.085	0.272 ± 0.020
14	α-Terpinolene	0.253 ± 0.019	0.358 ± 0.036	0.431 ± 0.030
15	β-Pinene	n.d.	0.370 ± 0.055	0.279 ± 0.024
16	β-Ionone	0.02052 ± 0.00053	0.283 ± 0.032	0.1423 ± 0.0045
17	Sabinene	0.072 ± 0.016	0.188 ± 0.028	0.1208 ± 0.0066
18	Decane	0.168 ± 0.010	0.104 ± 0.013	0.1212 ± 0.0088
19	β-Myrcene	n.d.	0.1152 ± 0.0082	0.212 ± 0.012
20	Carvone	0.02622 ± 0.00052	0.063 ± 0.010	0.0158 ± 0.0026

*SD* standard deviation, Mean ± SD of 3 measurements, Averages in rows marked with the same letters differ significantly (*P* < 0.05)

Due to the fact that terpenes are characterized by a similar structure, different chemical compounds belonging to this chemical class may have similar retention indexes. This makes it very difficult to correctly identify.

**Fig. 2:**
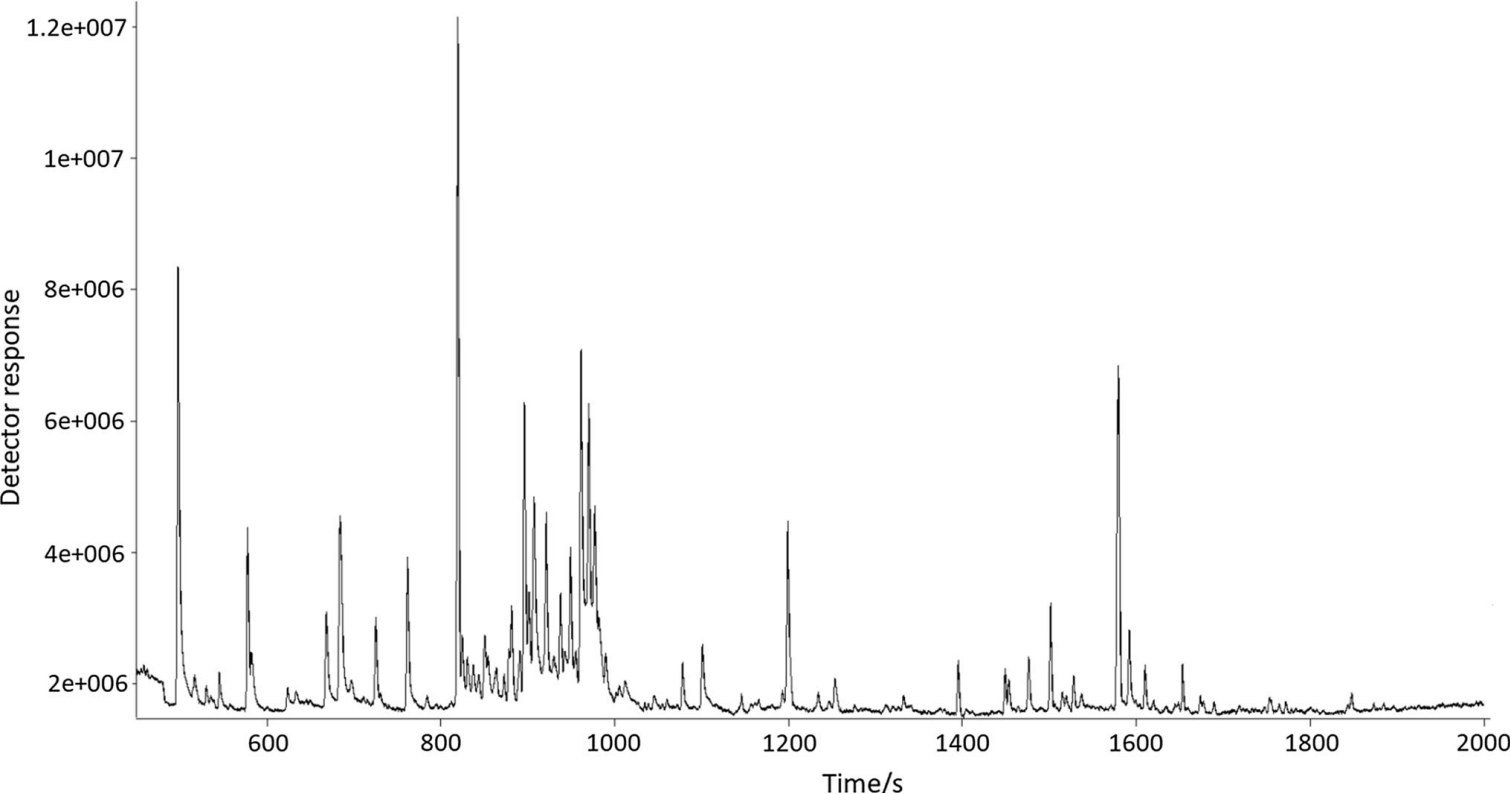
Chromatogram obtained by performing an analysis of limequat sample using GC-TOF–MS technique

To verify the correctness of tentative identification using the ultrafast GC, for the same fruit samples, analysis using GC–MS was carried out. A typical chromatogram obtained by analysing limequat sample with the use of GC-TOF–MS technique is presented in Fig. 2. In this apparatus, a long chromatographic column was used, which significantly extended the time of a single analysis (in the presented studies, the time of a single analysis was about 44 min), but also enabled the separation of analytes. The comparison of experimental spectra with the data included in mass spectra NIST 11 and Wiley 8 libraries was done to identify the chemical compounds. The value of similarity criterion was established to 850. Table 2 lists 20 major chemical compounds detected in the volatile citrus fractions using the GC-TOF–MS technique.

**Table 2:** The major compounds identified in the volatile fraction of kumquat, limequat, and lime using GC-TOF–MS technique

No.	Compound	Relative peak area mean ± SD/(%)		
		Kumquat	Limequat	Lime
1	Limonene	2.63 ± 0.15^a^	13.70 ± 0.54^a^	n.d.
2	δ-Elemene	2.64 ± 0.44^a^	5.87 ± 0.26^a^	n.d.
3	4-Terpinenyl acetate	n.d.	7.5 ± 2.2	n.d.
4	β-Myrcene	0.679 ± 0.046^a^	4.555 ± 0.080^a^	2.913 ± 0.050^a^
5	Alloocimene	1.62 ± 0.24^a^	3.59 ± 0.66^a^	0.03314 ± 0.00012^a^
6	Decanal	0.744 ± 0.048^a^	3.868 ± 0.057^a,c^	0.512 ± 0.036^c^
7	Bergamotene	n.d.	3.11 ± 0.21	n.d.
8	Caryophyllene	0.420 ± 0.046^a^	2.914 ± 0.023^a^	2.582 ± 0.043^a^
9	γ-Terpinene	1.246 ± 0.014^a,b^	2.440 ± 0.099^a^	6.14 ± 0.18^b^
10	Dodecane	0.813 ± 0.045^a^	2.5123 ± 0.0097^a,c^	0.519 ± 0.040^c^
11	Linalyl acetate	0.42 ± 0.18^a^	2.00 ± 0.33^a^	4.096 ± 0.030^a^
12	Ocimene	0.88 ± 0.74^a^	2.104 ± 0.080^a^	0.0618 ± 0.0015^a^
13	Fenchyl alcohol	n.d.	1.699 ± 0.017	n.d.
14	Bisabolane	n.d.	1.409 ± 0.025	n.d.
15	γ-Elemene	n.d.	1.348 ± 0.075	n.d.
16	2.6-Dimethylheptane	n.d.	1.193 ± 0.051	n.d.
17	α-Terpinolene	4.40 ± 0.40^a,b^	1.0968 ± 0.0036^a^	2.06 ± 0.11^b^
18	Cymenene	1.542 ± 0.025	0.987 ± 0.028	2.11 ± 0.16
19	β-Pinene	2.203 ± 0.052	0.413 ± 0.018	n.d.
20	Carvone	0.547 ± 0.060	0.3531 ± 0.0017	0.0989 ± 0.0082

*SD* standard deviation, mean ± SD of 3 measurements, averages in rows marked with the same letters differ significantly (*P* < 0.05)

Based on the chromatograms (Figs. 1 and 2), it can be stated, that GC–MS allows to detect a bigger number of chemical compounds, and thus the characteristics of the volatile profile of sample are more accurate.

Chemical compounds identified using both the GC-FID and GC–MS techniques, were 6 terpenes, namely: limonene, γ-terpinene, cymenene, β-pinene, β-myrcene, and carvone. These substances are characteristic for citrus fruit, e.g., kumquat [13, 14]. These compounds were detected in peel extracts of limequat samples by Casilli et al. [15]. Casilli et al. also identified other terpenes in the studied samples: α-terpinene, α-phellandrene, α-terpinolene, or sabinene. At least 10 of the chemical compounds presented in Table 1 were identified in previous literature reports.

To classify the samples due to their botanical origin analysis of variance ANOVA with Duncan’s new multiple range test was used. Average values for selected chemical compounds between three types of samples were compared. Based on the results obtained with the use of ultra-fast GC technique, it can be concluded that only for the chemical compound identified as 3-heptanone, all fruit samples differed statistically from each other. The analysis of chromatographic peak areas of signals corresponding to α-terpinene made it possible to distinguish kumquat samples from other citrus, and α-phellandrene lime samples from others. Data obtained with the use of GC–MS are more accurate (Table 2). Comparing the content of 5 substances in the volatile fraction makes it possible to classify samples of three fruit species. These substances were namely: β-myrcene, alloocimene, caryophyllene, linalyl acetate, and ocimene. Therefore, the use of targeted analysis would be justified in this case. In addition, it should be noted that in the case of ultrafast GC, direct injection of headspace was performed, while in the case of GC-TOF–MS, solid phase microextraction (SPME) was used to enrich and isolate analytes. The application of the isolation step may result in allowing only a part of the sample to be analysed, since the chemical compounds with the highest affinity to the stationary phase are adsorbed on the fibre. For this reason, the use of SPME is justified in the targeted analysis, while in the case of profiling and fingerprint, a direct injection is recommended to get a holistic information of the chemical composition of the sample.

Based on the obtained results, it can be concluded that the targeted analysis is sufficient to distinguish kumquat, limequat, and lime samples using the GC-TOF–MS technique. However, this approach has many limitations, such as a long analysis time, high cost of analyses and reagents, or the need to use standards to confirm the identification of detected compounds. In the case of the ultrafast GC technique, performing a targeted analysis is almost impossible. However, the solution to these limitations may be the use of fingerprinting coupled with chemometric methods.

### Fingerprint analysis

**Fig. 3:**
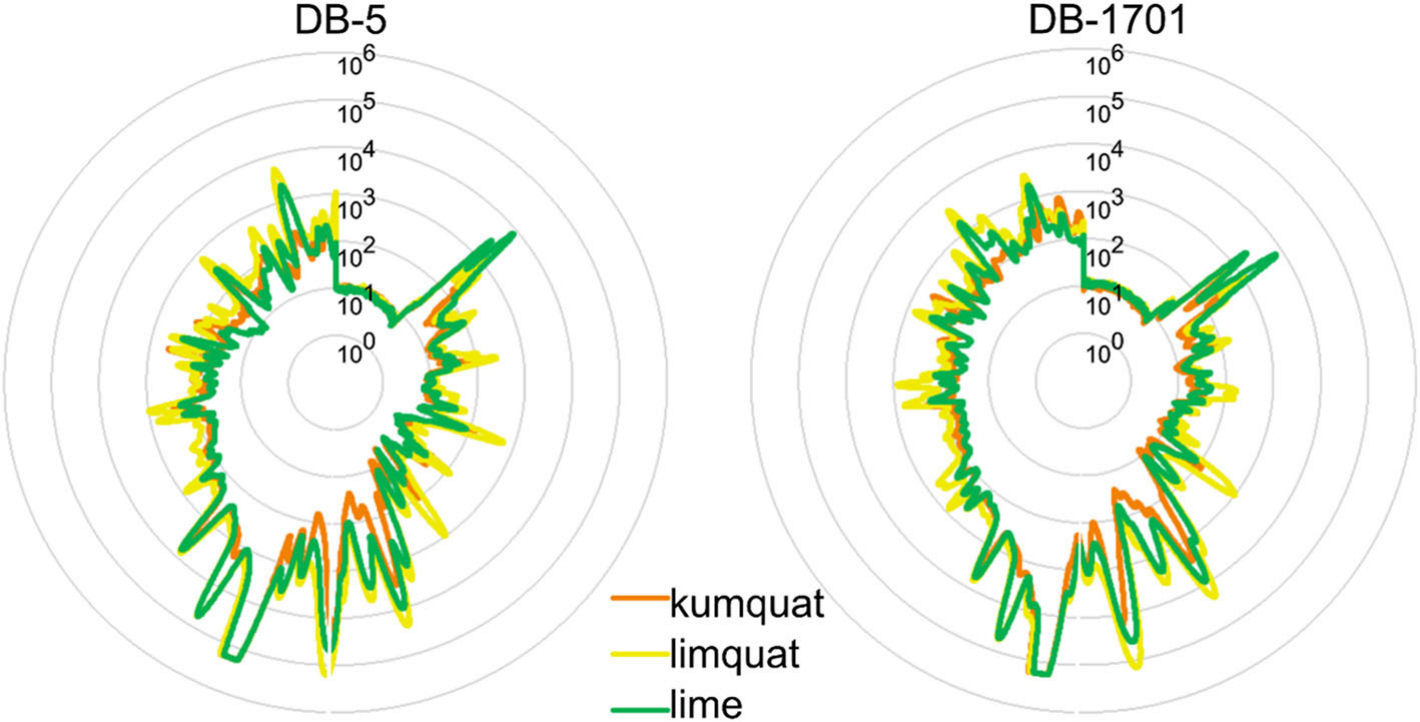
Graphical display of fingerprints as radar plots obtained on the basis of an analysis carried out using two chromatography columns

A fingerprint method is a mutual pattern of several common chemical compounds with some characteristics in all the analysed samples [16]. It is commonly used to distinguish food samples. Parastar et al. combined fingerprint analysis using GC–MS with chemometric to analyse secondary metabolites of citrus peels [17]. Fingerprint analysis using mass spectrometry and electronic nose based on sensors is also used for determination fruit maturity [18]. Previous reports show the difference in aroma of limequat and kumquat, what determines difference in their fingerprints [11]. Radar plots showing the fingerprint of three species of fruits are shown in Fig. 3. Radar plots are a very common way to show multivariate data in a simple manner [19].

The similarity between chromatographic fingerprints could not comprehensively reflect the similarity in the contents of a few important components. The chromatographic peaks are not sufficiently separated. One of the reasons could be relatively too short time of chromatographic analysis. Therefore, data interpretation cannot be based on individual chemical compounds, which is why a holistic approach was applied at work. It allows for analysis of the entire fragrance profile of the sample analysed [20, 21]. Based on the radar plots, it is not possible to clearly distinguish the tested samples. Therefore, it was necessary to conduct further statistical analysis.

### Principal component analysis (PCA)

Principal component analysis is one of the most commonly often used chemometric techniques. The main purpose of the PCA analysis is to extract the important information from the data set described by several dependent variables and to present them as a set of new orthogonal variables [22]. It is commonly used in fruit analysis [22]. It can be also used to assess the authenticity or detection of adulterations of food products, including fruit juices [23]. Principal component analysis was used to analyse citrus hybrid fruits before. Shaw et al. carried out an analysis to distinguish new grapefruit hybrids from parent fruits [24].

**Fig. 4:**
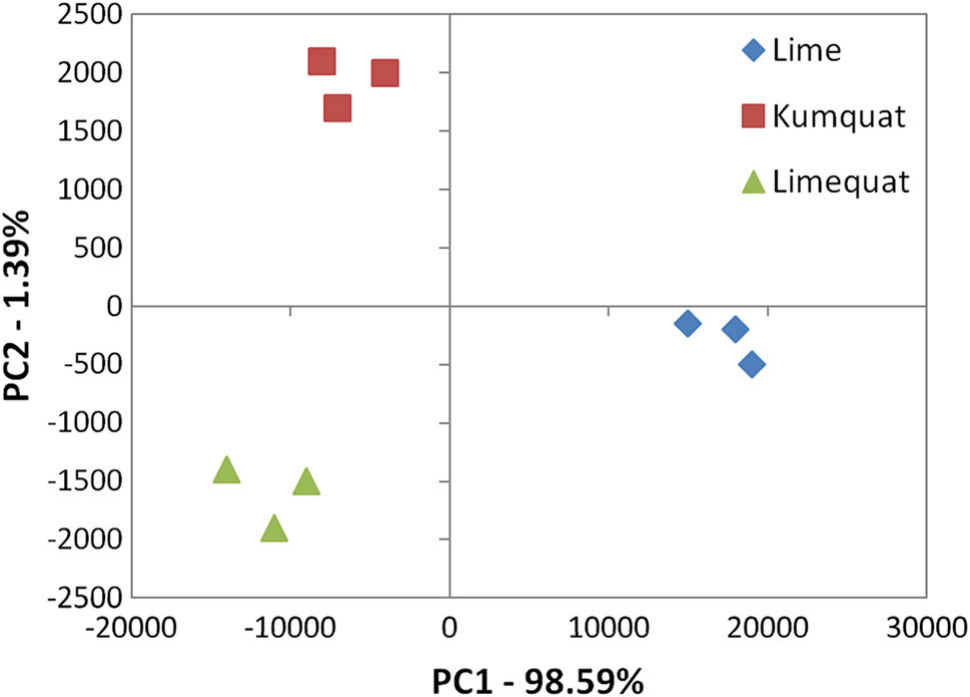
The result of the PCA analysis with the reference group of lime fruit, obtained as a result of fruit samples analysis (kumquat, limequat, and lime) using the Alpha M. O. S. Heracles device

As a visual display, the PCA for the interpretation of data obtained with the use of an electronic nose is shown in Fig. 4. As a result, three groups of points were obtained representing lime, kumquat, and limequat. The formed groups are clearly distinct from each other, which confirm the difference between them. Short distance between points among group representing one fruit species and obtained from different fruits can determine that their chemical composition is similar. It can be also concluded that lime could be distinguished from samples of other citrus fruits along PC1. However, samples limequat and kumquat were separated along PC2, so these two fruits have similar volatile composition. The PC1 and PC2 values are 98.59 and 1.39%, respectively. The sum of the first and second main components is 99.98% of the data variance. This means that the first two components explain nearly 100% of the total data volatility.

### Discriminant function analysis (DFA)

**Fig. 5:**
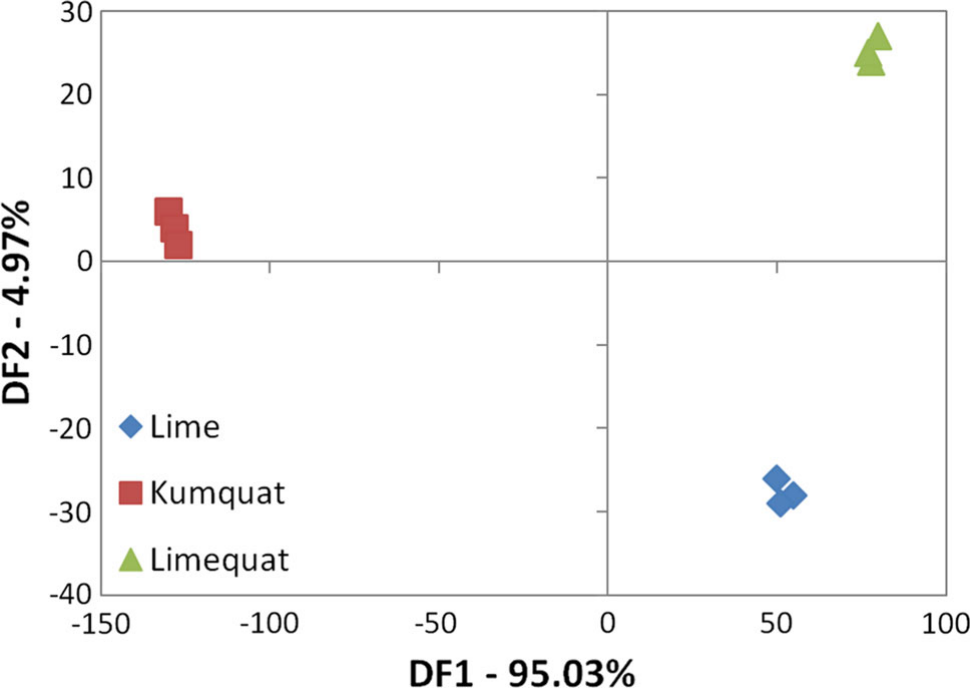
The result of the DFA analysis, obtained as a result of fruit samples analysis (kumquat, limequat, and lime) using the Alpha M. O. S. Heracles device

The basis for the discriminant function analysis is the creation of a function linearly dependent on the concentration of chemical compounds present in volatile fractions of the investigated fruit. DFA analysis allows the separation of data obtained into naturally occurring groups [25]. In contrast to PCA, DFA is used to distinguish groups not to present the actual data visualization [26]. Previous literature reports show that the discriminant component analysis can be a better option to distinguish between citrus hybrid fruits than PCA [27]. The results are more unambiguous. The resulting DFA analysis graph is shown in Fig. 5. The DF1 was accounted for 95.03%, the DF2 was accounted for 4.97% of among groups variability. DFA confirmed the difference in volatile profiles of hybrid and parent fruits accounting for a total variance of 100%. Root 1 separates the hybrid fruit from parent fruit. Moreover, according to DFA results, it can be stated that considering the fingerprint of analysed fruits, the volatile fraction of limequat is more similar to lime.

### Soft independent modeling of class analogies (SIMCA)

The SIMCA method is classified into multidimensional classification methods, which enables to model individual groups of samples. This method assumes that one sample can be classified in one, many or in a desired group. The SIMCA method is used in combination with the PCA model [25]. The use of principal component analysis and successive creation of multidimensional fields with clearly defined limits for individual groups allows for the unambiguous classification of unknown samples into individual groups.

**Fig. 6:**
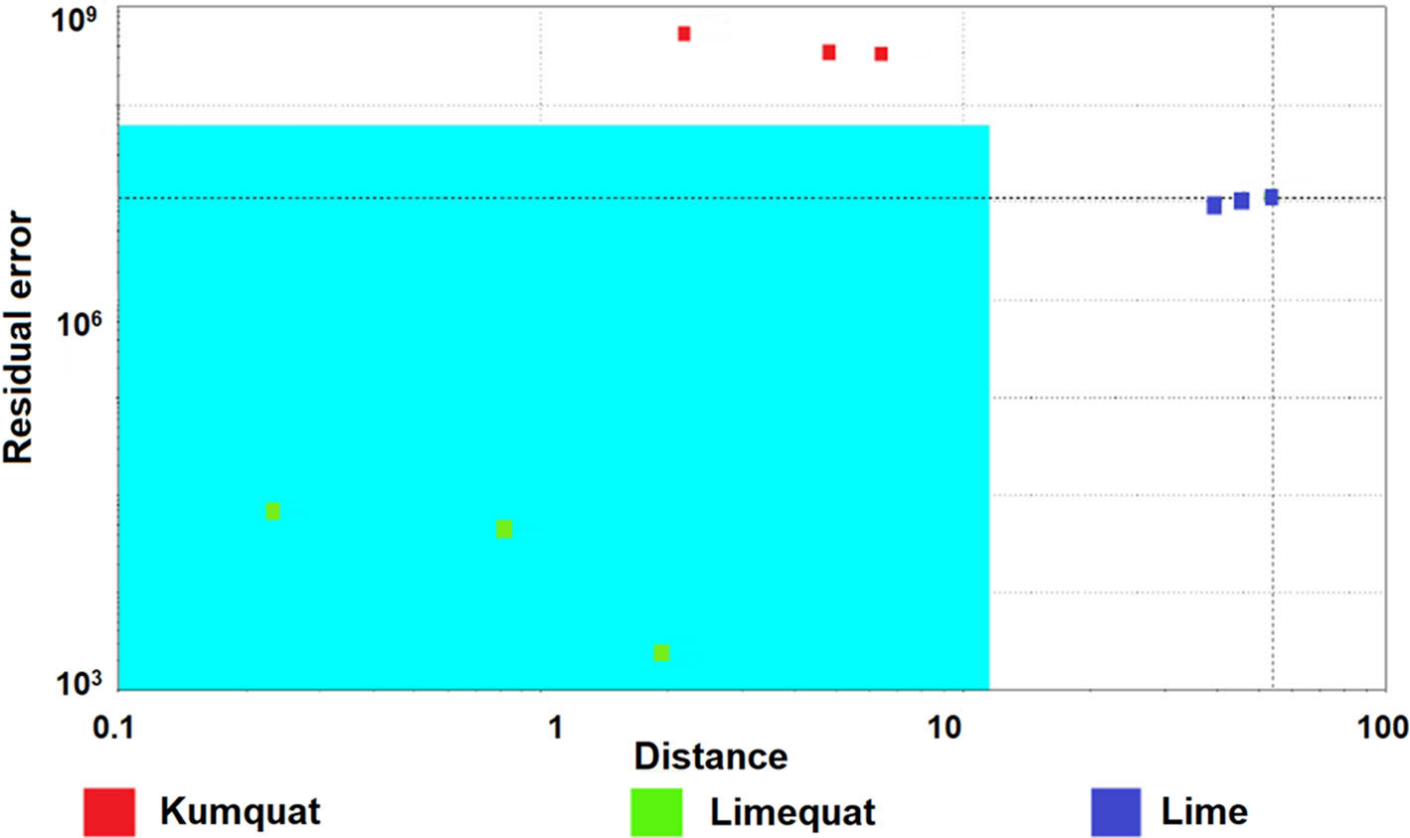
The result of the SIMCA analysis with the limequat reference group, as obtained from the analysis of fruit samples (kumquat, limequat, and lime) using the Alpha M. O. S. Heracles II device

**Fig. 7:**
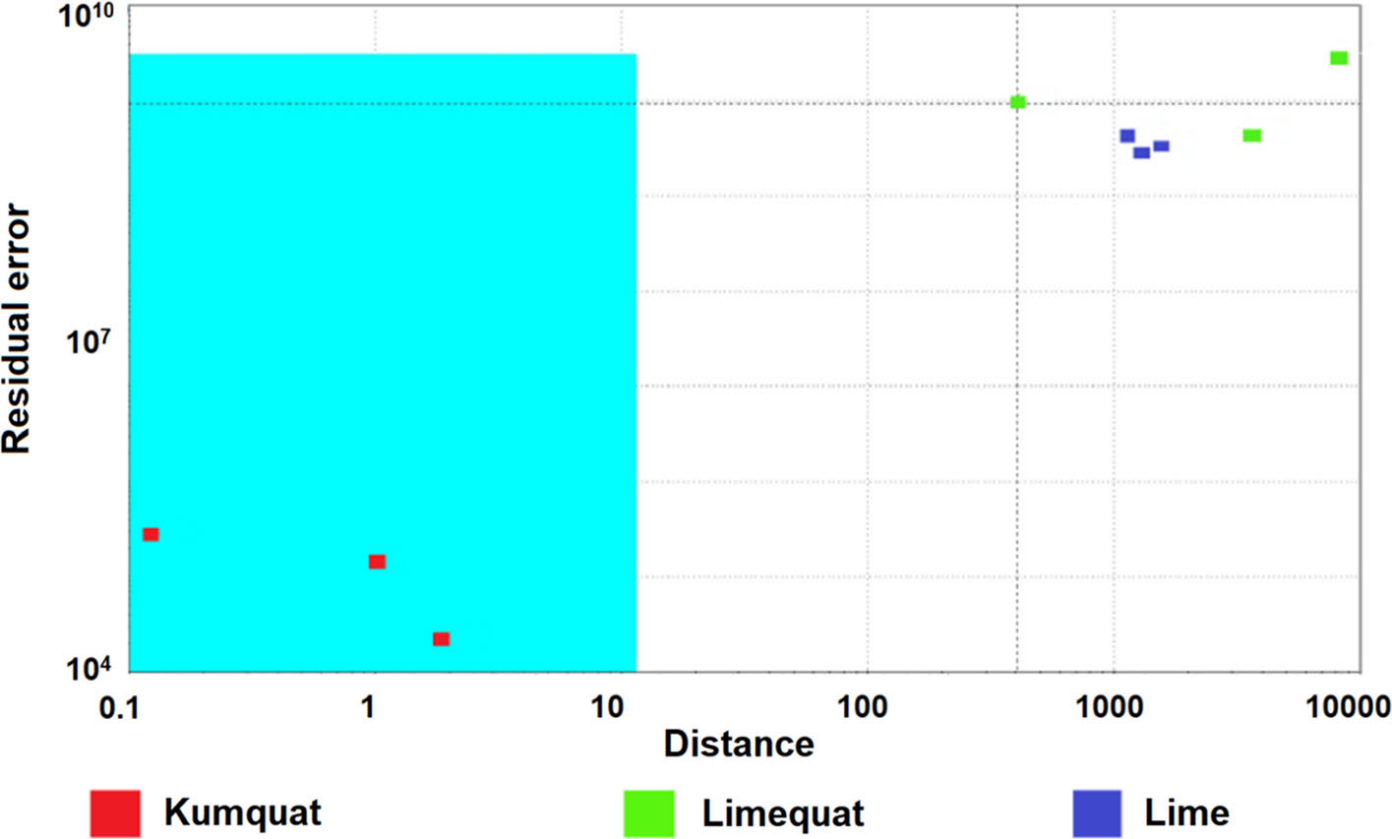
The result of the SIMCA analysis with the kumquat reference group, as obtained from the analysis of fruit samples (kumquat, limequat, and lime) using the Alpha M. O. S. Heracles II

**Fig. 8:**
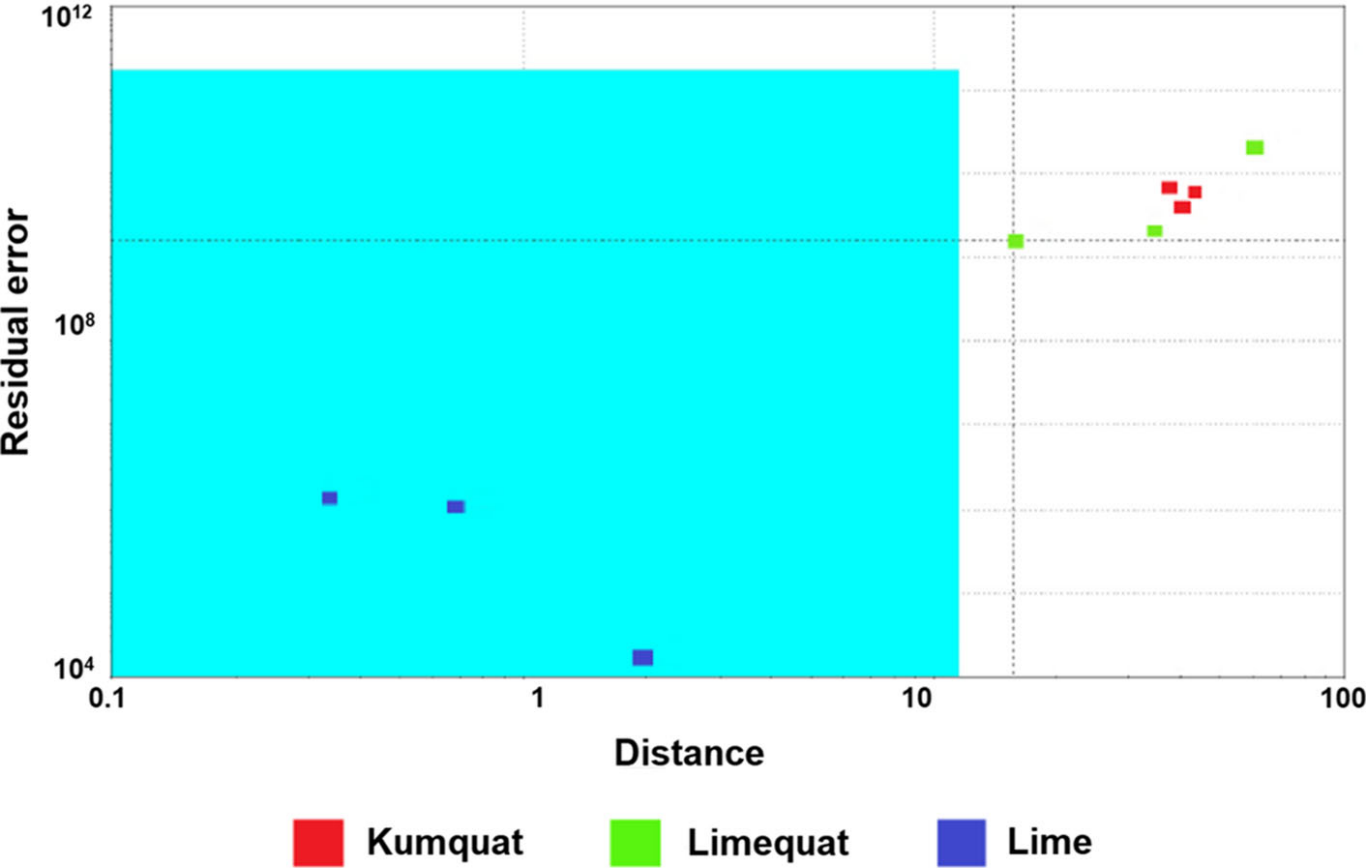
The result of the SIMCA analysis with the lime fruit reference group, as obtained from the analysis of fruit samples (kumquat, limequat, and lime) using the Alpha M. O. S. Heracles II

The result of the analysis allowed to distinguish the kumquat, lime, and limequat. The field marked in the chart is a confidence envelope. It is created so that the ends of the hyperplane of each class are closed off by setting statistical control limits along the retained principal components axes (i.e., score value between ± 0.5 times score standard deviation). If the points responding to individual samples are within the envelope, then the result is unambiguous and these samples belong to one group. Based on the SIMCA method, fruits can be distinguished. Figures 6, 7 and 8 present the results of the SIMCA analysis with the selected reference group of kumquat and lime fruit, respectively. The assumption of the model is that samples fulfilling certain criteria should be included in a specific area, called a confidence envelope. Figures 6, 7 and 8 show that thanks to the use of the electronic nose and the statistical SIMCA model, it is possible to distinguish the hybrid fruit from the parent fruits. In each of the following cases, another fruit was a reference sample when creating the model. In each case, only the points corresponding to the reference fruits are in the confidence envelope.

### Statistical quality control (SQC)

SQC analysis was employed to control the quality of fruit samples. SQC is a quick and easy statistical evaluation of the occurrence of sample differences in a given group of samples. Based on the results of the SQC analysis, sample elimination may be performed if it is significantly different from the other samples from the given group [28]. For this reason, it is used to control the quality of products on production lines or to control the entire process. As a result of using the SQC analysis, the samples are qualified for individual groups. Samples belonging to the appropriate classes should be placed in the appropriate area of confidence on the graph. This area is defined by appropriate parameters and bounded by upper and lower limits of these parameters which are set at ± 3 standard errors from the mean.

**Fig. 9:**
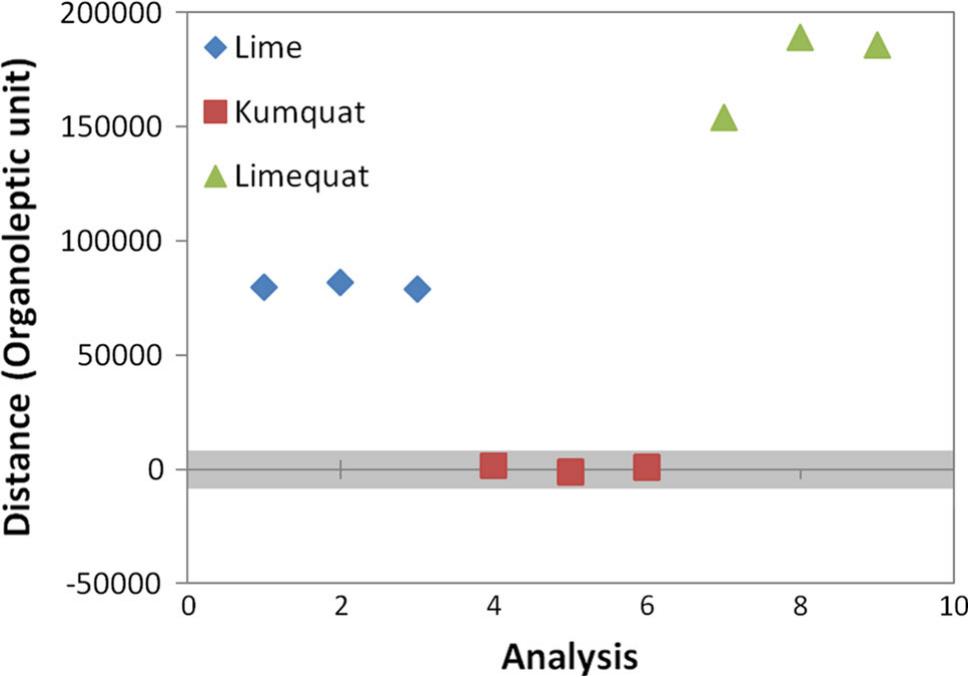
The result of the SQC analysis with the kumquat fruit reference group, as obtained from the analysis of fruit samples (kumquat, limequat, and lime) using the Alpha M. O. S. Heracles II

As a result of the SQC analysis, the graph shown in Fig. 9 was obtained. Based on it can be assessed that there is a noticeable difference in analysed fruit aroma, because the points corresponding to reference points, namely limequat are places within the confidence area. This is the proof of the distinction of the botanical sample of the hybrid limequat fruit from its parent fruits.

## Conclusions

In this paper, the usefulness of the electronic nose based on ultrafast GC for classification regarding to botanical origin of hybrid fruit samples, was presented. It was possible to evaluate the effectiveness of fast analysis of volatile fractions of fruits by comparing the results of analyses performed using the ultrafast GC-FID and GC–MS. It should be noted that any quality differences may be caused by different sampling methods. The use of ultra-fast GC has, therefore, proven this equipment is a tool proper for quick evaluation of the fragrance profile of fruit, providing a short analysis time. Applying a holistic approach, based on the analysis of the entire volatile fraction, without the prior separation of chemical compounds, seems to be an insufficient approach to the botanical distinction of limequat, kumquat, and lime fruit samples. The four chemometric models presented above are useful for assessing the botanical origin of the limequat hybrid fruit. It is possible to distinguish the analysed fruit samples by employing each of above described methods. Therefore, it is not necessary to carry out a work- and time-consuming process of identifying chemical compound by the use of GC–MS.

## Experimental

Three kinds of citrus fruit were used for analysis: kumquat (*Citrus japonica),* key lime *(Citrus *× *aurantifolia)*, and hybrid of those two—limequat *(Citrofortunella *× *floridana).* The fruits for analysis were purchased from local suppliers in Gdansk. Each fruit variety was tested with fruits purchased from three suppliers. Information of the origin of the fruit was obtained only from one supplier and they were: Israel in the case of limequat and kumquat and Brazil in the case of lime.

### Sample preparation

Fruits were washed in tap water, then rinsed with distilled water and then peeled. The samples were ground and homogenized in an agate mortar. 5.0 ± 0.1 g of homogenized sample of citrus fruit were weighted into the 20 cm^3^ glass vial and then 1 cm^3^ of deionized water was added. Vials were capped with a cap with Teflon-silicone membrane. In addition, each sample was prepared by homogenizing the pulp of 3 different fruits in an agate mortar, one sample corresponding to one supplier.

### Electronic nose analysis

The electronic nose based on ultra-fast gas chromatography Alpha M.O.S., (trade name Heracles II) was used to carry out the analysis. The system is equipped with an HS100 autosampler. The syringe was directed to injector working in splitless mode. Then, the sample was transferred to the sorption trap filled with 10 mg of sorbent Tenax^TA^. Components of the sample were thermally desorbed to two parallel chromatographic columns with different polarity (non-polar MXT-5 and medium polar MXT-1701) with a length of 10 m and an internal diameter of 0.18 mm. System is equipped with two micro flame ionization detectors (μFID), which can operate at temperatures up to 300 °C. The system contains AlphaSoft V12 software with implemented modules for chromatographic, chemometric and sensory analysis of characteristics of detected chemical compounds, the AroChemBase V4 HERACLES V12 library.

Hydrogen was used as a carrier gas. The process of incubation was carried out for 300 s in 80 °C. The vial with sample was stirred at 500 rpm in agitator. 2500 mm^3^ of the sample’s volume was injected for 15 s with the volumetric flow rate of the carrier gas flow equal to 250 mm^3^/s. The injector temperature was 200 °C and the pressures of carrier gas were set up at 250 kPa. Desorption of the sample lasted 20 s at the initial temperature of the trap sorption of 40 °C and the pressure of 80 kPa. The temperature of FID detectors was 270 °C.

### Gas chromatography–mass spectrometry analysis

Solid phase microextraction was utilized for the isolation and enrichment of analytes using divinylbenzene/carboxen/polydimethylsiloxane (DVB/CAR/PDMS)-coated fibre with thickness of 50/30 µm and length of 2 cm (Sigma-Aldrich). Extraction was done at 40 °C for 35 min. Next, the SPME fibre with extracted analytes was automatically transferred into the injector port of the gas chromatograph for thermal desorption for 6 min.

Separation and detection of the components of the volatile fraction of citrus fruit samples were done using Agilent 7890A gas chromatograph (Agilent Technologies, Palo, Alto, CA, USA), equipped with a single jet dual stage cryogenic modulator utilizing liquid nitrogen, coupled with Pegasus IV time-of-flight mass spectrometer (LECO Corp., St. Joseph, MI, USA). During the research chromatographic column with stationary phase Equity1 (Varian, Mississauga, ON, Canada) 30 m × 0.25 mm I.D. with 0.25 µm film thickness was utilized. Separation was achieved using GC temperature program for primary oven as follows: initial temperature of 40 °C held for 3.5 min then ramped to 250 °C at 6 °C min^−1^ and held for 5 min. Hydrogen was used as a carrier gas at a constant flow of 1.0 cm^3^ min^−1^. The total time of analysis was 43.5 min. The injector was operated in splitless mode at 250 °C. The transfer line and ion source were kept at 250 °C. Ions in the *m/z* = 40–400 range with data acquisition rate of 125 spectra/s were analysed.

### Data processing

In case of GC–MS results data processing was automatically done using the peak deconvolution algorithm included in the ChromaTOF-GC Software (LECO Corp., version 4.44.0.0). Tentative identification of the analytes was done through experimental spectra matching with the data included in NIST 11 and Wiley 8 mass spectral libraries. In case of electronic nose analysis, the set consists of dedicated AlphaSoft V12 software with implemented modules for chromatographic, sensory analysis of characteristics of detected chemical compounds, the AroChemBase V4 library. Chemicals were identified by comparing Kovat’s index with literature data.

### Statistical analysis

The peak areas obtained by the use of ultrafast GC-FID and GC-TOF–MS analyses were used for sample classifications. Means of the of three measures of peak areas were calculated to verify the statistical significance of the qualitative analysis results, In addition, for each average peak area of chemical compound, standard deviation was determined. Differences between groups were analysed using STATISTICA 12 (StatSoft, Inc., Tulsa, Oklahoma, USA). Two-way analysis of variance (ANOVA) followed by Duncan’s new multiple range test was carried out. P values less than 0.05 were considered to be significant.
